# Analysis and Design of a CMOS Ultra-High-Speed Burst Mode Imager with In-Situ Storage Topology Featuring In-Pixel CDS Amplification

**DOI:** 10.3390/s18113683

**Published:** 2018-10-30

**Authors:** Linkun Wu, David San Segundo Bello, Philippe Coppejans, Jan Craninckx, Andreas Süss, Maarten Rosmeulen, Piet Wambacq, Jonathan Borremans

**Affiliations:** 1 Imec, 3001 Heverlee, Belgium; David.SanSegundoBello@imec.be (D.S.S.B.); Philippe.Coppejans@imec.be (P.C.); Jan.Craninckx@imec.be (J.C.); Andreas.Suess@imec.be (A.S.); Maarten.Rosmeulen@imec.be (M.R.); Piet.Wambacq@imec.be (P.W.); Jonathan.Borremans@imec.be (J.B.); 2 Department of Electronics and informatics (ETRO), Vrije Universiteit Brussel, 1050 Brussels, Belgium

**Keywords:** image sensors, in-situ storage, ultra-high-speed imaging, burst mode, million frames per second, in-pixel amplification

## Abstract

This paper presents an in-situ storage topology for ultra-high-speed burst mode imagers, enabling low noise operation while keeping a high frame depth. The proposed pixel architecture contains a 4T pinned photodiode, a correlated double sampling (CDS) amplification stage, and an in-situ memory bank. Focusing on the sampling noise, the system level trade-off of the proposed pixel architecture is discussed, showing its advantages on the noise, power, and scaling capability. Integrated with an AC coupling CDS stage, the amplification is obtained by exploiting the strong capacitance to the voltage relation of a single NMOS transistor. A comprehensive noise model is developed for optimizing the trade-off between the area and noise. As a proof-of-concept, a prototype imager with a 30 µm pixel pitch was fabricated in a CMOS 130 nm technology. A 108-cell memory bank is implemented allowing dense layout and parallel readout. Two types of CDS amplification stages were investigated. Despite the limited memory capacitance of 10 fF/cell, the photon transfer curves of both pixel types were measured over different operation speeds up to 20 Mfps showing a noise performance of 8.4 e^−^.

## 1. Introduction

Ultra-high-speed imaging has been proven to be a very useful observation technique for applications where the analysis of fast transient events is required, such as material study and testing, failure dynamics, combustion research, and medical research [1,2,3,4]. The first commercial high-speed imager, Kodak Ektapro EM, provided the capability of capturing frames of 240 × 192 pixels at a speed of 2000 frames per second in 1989 [5]. Nowadays, thousand-frames-per-second (kfps) video imagers are widely used for broadcast and even show up in consumer products, such as cell phones [6]. Therefore, mega-frames-per-second (Mfps) and beyond has become a new playground for researchers. For Mfps imagers with resolutions of 1 megapixel or higher, the practical speed limitation is the off-chip data throughput which makes the continuous readout infeasible.

To solve the readout throughput bottleneck due to the high frame rate and high spatial resolution, the rotating mirror imagers [7] were developed achieving high frame rates up to 25 Mfps. These imagers redirect the time series of light information (frames) into multiple cameras. As a more integrated solution, streak cameras first convert the incident light into electrons, and different time stamped information is then separated by applying an electric field [8]. Meanwhile, the encoding and storing of time information in the spectral domain was also investigated in order to relax the speed on the imagers [9]. Furthermore, by exploiting the sparsity in the images, compressive sensing was applied, demonstrating that high speed images can be measured and reconstructed with the help of multiple apertures without using expensive optics [10]. More techniques such as holography and FRAME imaging have been exploited to capture high speed phenomena [11,12].

All those ultra-high-speed imaging techniques are only indirectly solving the problem of slow imager sensors at the cost of expensive optical setups or heavy post-processing. In the domain of Mfps, burst mode imagers appear to be a more straightforward solution. In burst mode operation, a large amount of image data (frames) is captured at high speed, stored on the chip temporarily, and read out at feasible speeds afterwards. The number of frames stored in a single burst readout is referred as “frame depth.” Together with the operation speed and the noise performance, they are the most important specifications for burst mode imagers. Since the existing photosensitivity devices can already reach nanosecond speed [5], to further improve the frame rate and high frame depth, more work needs be done in the readout and storage circuits.

As the on-chip memories are mainly implemented in the analog domain, burst mode imagers can be categorized into CCD (charge domain) or CMOS (voltage domain) type, based on the implementation of the frame storage. Note that, in the scope of this paper, the term “CCD” is not linked to the CCD manufacturing process but to the fact that the storage units are operated in a traditional CCD manner. The collected photogenerated charges are stored and manipulated with potential pockets created by gate voltages. With a dedicated CCD process, two 16 Mfps imagers have been demonstrated with frame depths of 117 and 139 each [13,14]. Furthermore, a 180-frame-depth 5 Mfps CCD type imager was developed in a CMOS-compatible process with 800 µV noise [15]. However, CCD devices are typically power hungry due to the large switching power for charging and discharging the high capacitance of the CCD gates [16]. CCD imagers typically require high driving voltages, which leads to high power consumption. Since the switching power is proportional to the frequency, it is more difficult to achieve high operation speeds.

As an alternative, CMOS-type burst mode imagers first convert photogenerated charges into voltages and then store the voltage information in analog capacitors. As generic benefits from CMOS technology, the high integration capability allows many more circuits such as ADCs or other signal processing units to be integrated on chip, reducing the overall power of the imager system as well as the cost. Moreover, CIS (CMOS Image Sensor) technology has a dominant market share against their CCD competitors. As a mainstream technology, it can certainly provide a higher availability and accessibility. CMOS-type storage has several unique merits over CCD type storage. First, only very few switches need to be toggled for every storage in the voltage domain, while for storage in the charge domain all charge on the same storage chain always needs to be shifted. Thanks to the lower switching power consumption, the total power consumption is lower compared to the CCDs. Secondly, the CMOS supply voltage is easier to be optimized or reduced according to the actual signal swing, whereas CCD gate driving voltages are constrained by the voltage barrier requirements [17]. Thirdly, CMOS imagers can have region of interest (ROI) readout and random access of the pixels, while CCDs do not allow it. Using CMOS technology, researchers have successfully explored CMOS burst mode imagers with in-pixel memory [18,19,20]. Meanwhile, the external memory bank architecture is also demonstrated in a 20 Mfps 400 × 256 pixels imager [21]. As an improved version, the frame depth is further improved to 480 frames with customized 3D trenched memory cells [22]. Together with 3D stacking potential, we believe that CMOS technology is a promising technology for large format burst mode imagers, and this is the focus of this paper.

The main design challenge for burst mode imagers is to reach high frame depth, but that should not be achieved at the cost of high sampling noise. First, floating diffusion (FD) reset operation generates reset noise, which can be eliminated with correlated double sampling (CDS). To realize CDS, typically both the reset signal and video signal are stored and then subtracted from each other. Unlike charge domain storage, CMOS burst mode imagers suffer from the fact that the reset operation is done at the beginning of the signal chain during each acquisition, which demands a fast reset operation to be implemented for every pixel at the shutter speed. Even worse, as every pixel has multiple frames to store, conventional CDS implementation becomes extremely expensive in terms of area for CMOS burst mode imagers. Secondly, the kTC noise from the memory storage is another obstacle hindering the increase of the frame depth. Since the voltage domain memory storage will introduce additional sampling kTC noise, directly shrinking the memory cell is not a sustainable solution. Additionally, due to the fixed overhead from the required switches, the effective capacitance per memory cell will decrease with the increasing number of memory cells. Eventually, it sets a practical limit on the minimum dimension of the memory cell and the frame depth.

To solve the trade-off between high frame depth and low noise operation, based on the in-situ storage topology, we demonstrate a novel pixel architecture in this paper featuring in-pixel CDS amplification. The paper is organized as follows. In Section 2, the high-level structure of the proposed architecture is presented. System level trade-offs are discussed for each block. In Section 3, the circuit implementation and its operation are described in detail along with a comprehensive noise model, quantitively showing the noise improvement. Section 4 explains the design and layout of the test chip. The measurement setup and results are also discussed. Finally, the summary of the specifications and conclusions can be found in the Section 5.

## 2. Pixel Architecture

**Figure 1:**
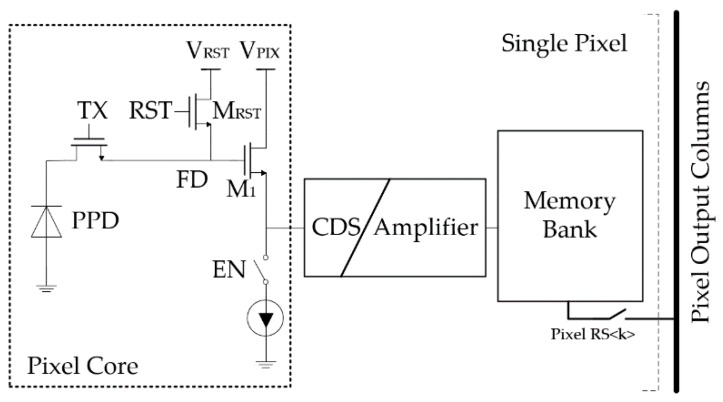
The proposed pixel architecture for burst mode imagers.

As mentioned, CMOS burst mode imagers suffer from heavy kTC noises from various sources and increasing the frame depth will lead to the deterioration of the noise performance. To improve the trade-off with a new design freedom, based on the in-situ storage topology, we propose a novel pixel architecture as shown in Figure 1. It consists of three basic parts: a photosensitive structure, referred as the pixel core based on the 4T Pinned Photodiode (PPD), a CDS amplification stage, and a local memory bank.

Because the memory bank requires the highest area budget, its placement has the most significant impact on the pixel architecture. First of all, although the external storage topology allows dedicated optimizations for the pixel core and the memory bank, such as better shielding, improved QE or fill factor [21], the in-situ storage topology is preferred here because it is better suited for a larger image format (e.g., 1 Mega pixels) based on the following reasons:

1. Low power consumption. External storage requires long signal lines running from the pixels over the pixel array to the memory at high speeds. As a result, a high capacitive load needs to be driven at high speed from each pixel, which is very challenging and power hungry. Moreover, this lengthy line is more likely shared among several pixels due to layout constraints. Consequently, the signal line must be multiplexed at even faster speed, thus making the speed problem even more critical.
2. Better area efficiency. For the high-speed applications, due to the short integration time (e.g., a few nanoseconds), limited by the light source, a larger pixel pitch is typically required for sufficient photon collection. Implementing the memory bank inside the pixel will maximize the utilization of the empty space, which is beneficial for reducing the total imager size.

Obviously, the major disadvantages for in-situ storage topology are the low fill factor and low QE. Fortunately, these negative impacts can be largely attenuated using the backside illuminated (BSI) technology [5,23]. The typical CMOS memory bank can consist of multiple analog memory cells as well as the peripheral switches for the row and column addressing.

The next important block is the photosensitive device and its peripherals. Like a conventional 4T pixel, the pixel core contains a PPD, a transfer gate, a reset transistor as well as a source follower (SF) M_1_ with an in-pixel current source for global shutter operation. The current source is made switchable allowing low power operation during the long readout time from the memory bank. Although the design of fast PPDs is outside the scope of this paper, the speed of photosensitive device is not necessarily the bottleneck of the system. The charge collection and transfer time has been reported in the range of a few nanoseconds [5,24,25]. The PPD is favored here primarily due to its low FD node capacitance, leading to high conversion gain (CG). The high CG can improve the sensitivity in the short integration time scenario, especially under low light condition. Moreover, in terms of input referred noise reduction, it is most effective to place the gain at the beginning of the signal chain.

However, there is no free lunch. Since the FD node is sized for high CG by making its capacitance small, the corresponding FD node reset noise is also increased, which dominates the total noise if no measures are taken. For instance, with a typical CG gain of 100 µV/e^−^, the FD node reset noise alone contributes more than 10 e^−^ input referred noise. Therefore, CDS is essential to reach low noise, e.g., less than 10 e^−^. Unfortunately, as already discussed, it is very area expensive to implement the conventional CDS in CMOS burst mode imagers.

To address this problem, we use in-pixel AC-coupled CDS [21,22,26]. An auto-zeroing capacitor $C_{\mathrm{CDS}}$ is inserted after the pixel SF M_1_, which serves as a high pass filter to remove the reset voltage at the FD together with the reset kTC noise. Because the in-pixel CDS is done prior to the storage operation, only one memory element per frame is needed versus two in the conventional CDS case. Assuming that the reset noise is completely canceled by the CDS operation, the total pixel noise will be mainly limited by the kTC noises from the sampling operation at the CDS and the memory storage, which can be modeled as

(1) $$V_{n,\mathrm{In}-\mathrm{pixel}\;\mathrm{CDS}}^{2}=\frac{kT}{\alpha \;C_{\mathrm{TOT}}}+\frac{kT}{\left(1-\alpha \right)\;C_{\mathrm{TOT}}/N},$$

where k is Boltzmann’s constant, T is the absolute temperature, $C_{\mathrm{TOT}}$ is the total capacitance budget per pixel for a fixed pixel pitch, N is the frame depth, and finally $\alpha \;C_{\mathrm{TOT}}=C_{\mathrm{CDS}}$ accounts for the overhead of $C_{\mathrm{CDS}}$ implementation. Therefore, the first term on the right-hand side of the equation represents the kTC noise from the CDS operation when sampling the reset signal. Since each memory cell has a capacitance of $\left(1-\alpha \right)\;C_{\mathrm{TOT}}/N$, the second one reflects the sampling noise due to the signal storage after the CDS.

As a comparison, the total pixel noise of the conventional CDS case is composed of the kTC noise from both reset signal and video signal sampling (2N memory cells are need). When the capacitance of the reset signal capacitor is equal to that of the video signal capacitor, the optimal noise can be achieved as

(2) $$V_{n,\;\mathrm{Conv}\;\mathrm{CDS}}^{2}=\frac{kT}{C_{\mathrm{TOT}}/\left(2N\right)}+\frac{kT}{C_{\mathrm{TOT}}/\left(2N\right)}.$$

**Figure 2:**
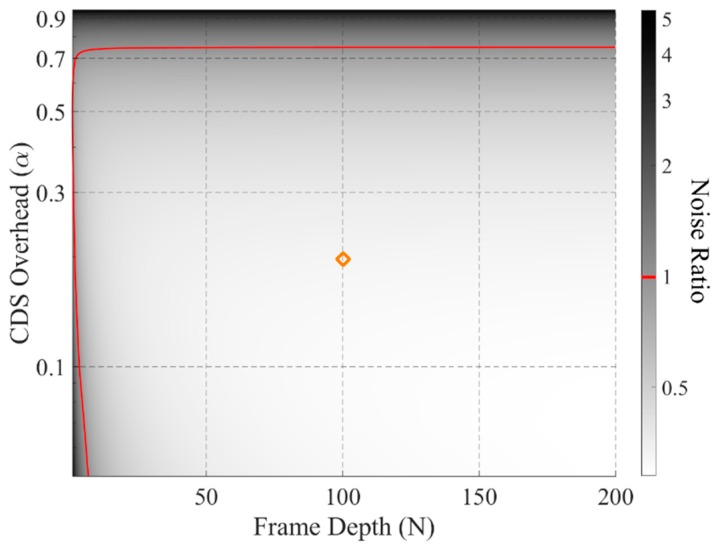
Noise power ratio between the in-pixel correlated double sampling (CDS) case and the conventional case as a comparison. E.g., given *α* = 0.2, *N* = 100, the ratio is 0.325 as highlighted, which means there is 67.5% noise reduction in the in-pixel CDS case compared to the conventional case.

Based on Equations (1) and (2), the output noise power ratio between in-pixel CDS and the conventional CDS, $ratio=V_{n,\mathrm{In}-\mathrm{pixel}\;\mathrm{CDS}}^{2}/V_{n,\mathrm{Conv}\;\mathrm{CDS}}^{2}$, can be evaluated versus the frame depth (N) and CDS overhead (α). The result is presented in Figure 2. As expected, when the CDS capacitance dominates (α close to 1), the noise performance is worse than that in the conventional case. Meanwhile, if the frame depth is too small, it is not worthy to use in-pixel CDS in terms of noise as it becomes a major noise limiting factor. Fortunately, for burst mode imagers where large N is required, we can exploit the quadratic relationship between the kTC noise and the corresponding sampling capacitance.

When FD node reset noise is attenuated by the in-pixel CDS technique, the sampling kTC noise from the memory bank can become the new dominant noise source. To cope against it, we propose implementing an additional in-pixel gain stage prior to the signal storage. As a typical measure to improve the input referred noise performance, the added gain will suppress not only the sampling noise from the memory bank but also that from following blocks. Moreover, higher gain also improves the original small signal swing [22], which is inherited from low light operation and short integration time. Especially for burst mode imagers, there are more buffering stages required due to the memory bank. Since it is difficult to implement the buffers as unity gain buffers inside the pixel, the extra amplification on the readout chain is desirable to compensate the gain loss. Another important benefit is that the leakage from the memory node is less pronounced, since the signal has been amplified in the voltage domain before storing it. Thus, the parasitic light sensitivity (PLS) due to a small capacitance can be improved.

**Figure 3:**
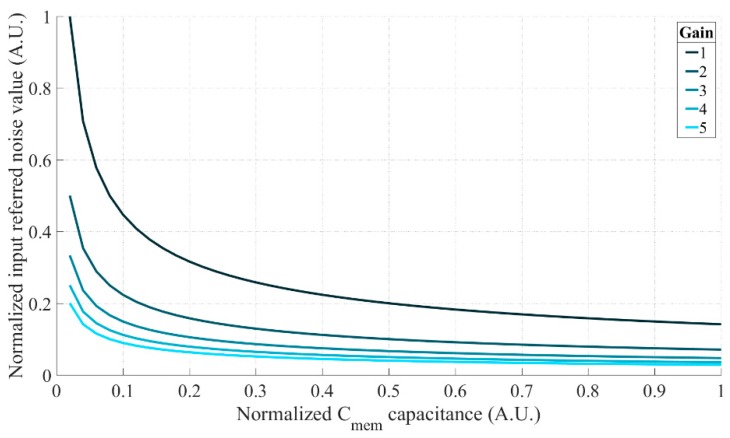
The input referred memory sampling noise against the capacitance value w.r.t. the amplification gains varying from 1 to 5.

However, limited by signal swing the readout circuitry can handle, it is not always feasible to have a large gain. To visualize the effectiveness of the noise reduction, the relationship between the input referred sampling noise of the memory bank and the amplification gain is investigated as shown in Figure 3. Since the input referred memory sampling noise power is reduced by the square of the amplifier gain, the amount of noise reduction quickly drops for large gains. Thus, considering the 1-volt signal swing, a gain of 3 is found to be a good balance between the noise and signal swing. Moreover, due to the inversely proportional relationship between the kTC noise and the memory capacitance, the noise performance can only be significantly improved at a dramatic cost in area. It implies an important trade-off between the memory bank and the amplifier that instead of enlarging the memories, introducing an amplifier is proved to be a more efficient way to reduce the input referred noise.

For these above reasons, the in-situ storage with in-pixel CDS amplification topology is believed to be a good match for burst mode imagers. However, implementing an amplifier inside the pixel is very challenging. One of the main limitation factors is the available area. Although the pixel pitch is relatively large (up to a few tens of micro-meters), considering the CDS structure and the large memory bank, there is little room left for implementing the pixel-level amplification. Secondly, the sharing techniques such as time-interleaving are not feasible for ultra-high-speed global shutter imagers. Due to the time constraints, each pixel must have its own dedicated amplifier, so the power also becomes a major concern. Lastly, because the N-type well may attract the photogenerated charges PMOS devices are avoided inside the pixel. Thus, we have to design the circuit using only NMOS devices.

Research has already been done on pixel-level amplification. First, the gain can be implemented as high conversion gain at the photosensitive devices [27,28,29,30]. These solutions are very area- and power-efficient, but it is very difficult to manipulate the conversion gain as it is limited by the FD parasitics. Additionally, those approaches typically require dedicated pixel design with complex technology processing. Secondly, pixel level circuit implementations for increasing the gain have been presented in [31,32,33,34,35]. Multiple stage amplifiers require large space and significant power and in the common-source open-loop amplifier gain is difficult to control. When implemented as an array, it is difficult to achieve a good gain pixel uniformity across the pixel array. Therefore, to tackle those problems, a single NMOS-only passive amplifier is chosen for this implementation. As a proof of concept, a prototype imager was developed, which will be discussed in detail in Section 3.

## 3. Prototype Implementation

Based on the proposed architecture, given a 30 µm pixel pitch, a prototype imager was developed targeting the recording of 108 frames at 20 Mfps with less than 10 e^−^ noise. In the test imager, two types of pixel variations were implemented. They share the same pixel core and the same memory bank but are equipped with two flavors of CDS amplification stages (referred to as Type A and Type B). Starting from common blocks, the detailed design of both variations will be described, and corresponding analytical noise models are then developed for design optimization.

**Figure 4:**
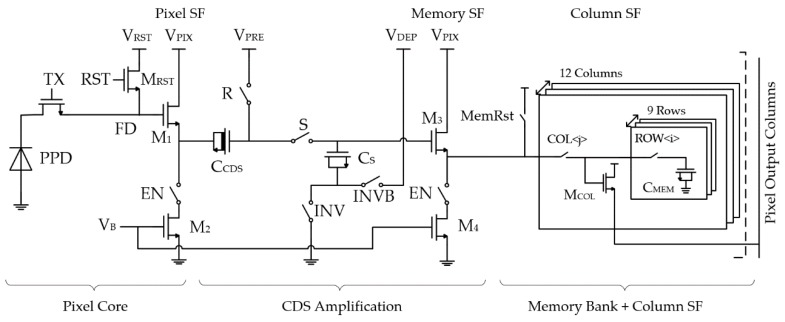
Pixel schematic (Type A).

The pixel schematic of Type A is shown in Figure 4. It consists of a 4T pixel core, a memory bank, and a CDS amplification stage in between. As mentioned, the 4T pixel core is preferred due to its high conversion gain for the low noise operation. A conversion gain of 100 µV/e^−^ at FD node is targeted in the design. To ensure an efficient charge collection and complete transfer at high speed, this pixel core requires a great deal of design effort and engineering [24], which is however outside the scope of this paper. In the test imager, the 4T pixel core used was not optimized for speed.

### 3.1. In-Situ Memory Bank with the Parallel Readout and Memory Reset

Both Type A and Type B pixels share the same in-situ memory bank consisting of 9 rows of 12 columns of memory cells as shown in Figure 4. Each memory cell contains only one switch and one NMOS capacitor to maximize the capacitance density. Limited by the physical design rules, reducing the memory cell size is not very effective in increasing the frame depth. In our current design, the switches are already comparable in size to the memory cells. Further shrinking the memory cell will not efficiently increase the frame depth but largely jeopardize the noise performance. Therefore, taking the parasitic capacitances into account, 10 fF/cell is chosen.

Memory cells are arranged in 9 rows and 12 columns inside the 30 µm pitch pixel to create a memory array. The column SF M_COL_ is shared among memory cells for each column, allowing the 12-column parallel readout. The reuse of M_COL_ introduces unwanted charge sharing among M_COL_, the parasitics, and the memory capacitor. Fortunately, this gain loss can be compensated by the extra amplification, showing the superiority of the proposed topology. Additionally, the in-situ configuration minimizes the parasitics of the potential column line, which is again beneficial for maintaining the gain. To prevent the imager lag from the previously read memory cell due to the same charge sharing, the switch MemRst is added to reset the gate of M_COL_ before reading the next memory cell.

The 9 × 12 memory bank is operated as follows. During the writing phase, the memory cells are filled up first along the columns within the current row, and this is then repeated for the all the columns in the next row until all memory cells are written. During the readout, the switches MemRst and COL are first ON to reset the gate of the column SF M_COL_ and later the corresponding ROW signal is turned ON, triggering the parallel readout of the memory cells from the same row.

### 3.2. CDS Amplification

**Figure 5:**
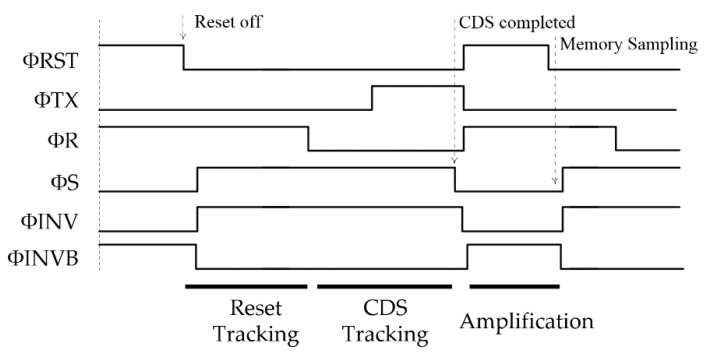
Pixel operation timing for both Type A and Type B (pipeline version).

The control signals’ timing is the same for Type A and Type B. The detailed operation of the in-pixel CDS amplification is presented in Figure 5. The CDS operation begins with resetting the FD node via RST transistor M_RST_. At the same time, the other side of $C_{\mathrm{CDS}}$ is set to $V_{\mathrm{PRE}}$ by keeping the switch R ON. During R is ON, the switches S and INV are turned ON resetting $C_{S}$. Secondly, the reset tracking phase is initialized when RST goes OFF and ends when R is turned OFF after the clock feedthrough settles. Thus, the reset voltage including the kTC noise $V_{n,\;\mathrm{FD}}$ is buffered via M_1_ and sampled over $C_{\mathrm{CDS}}$ against the clamping voltage $V_{\mathrm{PRE}}$.

This reset tracking phase is very important and the most time critical for the AC coupling CDS as a high load $C_{\mathrm{CDS}}$ must be driven by the small pixel SF M_1_, which requires a careful design balance between the noise and power. Otherwise, if the reset is not sampled properly, it will leave some reset noise residue at the output.

When the transfer TX gate closes, the photon charge is converted into a voltage at the FD node, any further FD node voltage drop $V_{\mathrm{SIG}-\mathrm{RST}}$ will then pass through $C_{\mathrm{CDS}}$ as an AC coupled signal. Finally, the CDS operation is completed when the switch S is OFF. As a result, the single-ended CDS voltage of $V_{\mathrm{PRE}}-A_{\mathrm{PIX},\mathrm{SF}}\cdot A_{\mathrm{CDS}}\cdot V_{\mathrm{SIG}-\mathrm{RST}}$ is sampled over $C_{S}$ with respect to the ground, where $A_{\mathrm{PIX},\mathrm{SF}}$ represents the pixel SF stage gain, and $A_{\mathrm{CDS}}$ represents the gain of the CDS stage. At complete settling, there is no presence of $V_{n,\mathrm{FD}}$ in the CDS signal.

The CDS operation comes with a gain loss, since $C_{\mathrm{CDS}}$ and $C_{S}$ form a capacitive voltage divider, which has the gain $A_{\mathrm{CDS}}=C_{\mathrm{CDS}}/\left(C_{\mathrm{CDS}}+C_{S}\right)$. To make this CDS useful, $C_{\mathrm{CDS}}$ should be much greater than $C_{S}$ to avoid excessive gain loss. Both $C_{\mathrm{CDS}}$ and $C_{S}$ are implemented as MOSFET capacitors to maximize the capacitance density. Additionally, due to the high reset voltage level required by the PPD, $C_{\mathrm{CDS}}$ is implemented as a depletion mode NMOS transistor, which can stay in the inversion mode even with a small gate-source voltage allowing a stable capacitance.

Once the CDS signal is sampled on $C_{S}$, the FD node can start resetting for the next frame in a pipeline fashion. A key aspect of the pixel circuitry presented here is that it provides amplification of the CDS signal before storage in the capacitor array, so the noise caused by the small memory capacitance can be attenuated. Within the limitations of the CIS technology, the passive single transistor amplifier is chosen [36], consisting of only one MOSFET capacitor $C_{S}$ and two switches INV and INVB. Applying non-overlapping complementary signals on INV and INVB, the voltage gain is obtained by toggling the shorted source and drain voltage of $C_{S}$ from ground to a specific DC voltage $V_{\mathrm{DEP}}$. The gain is yielded by taking advantage of the intrinsic capacitance–voltage (CV) characteristic of the MOS transistor: when the transistor is biased in the inversion region, the capacitance value of $C_{S,\mathrm{INV}}$ can be several times greater compared to the capacitance $C_{S,\mathrm{DEP}}$ in the depletion region. By forcing the transistor moving from inversion mode into depletion mode, because of the charge conservation on $C_{S}$, a voltage gain $A_{\mathrm{AMP}}$ of $C_{S,\mathrm{INV}}/C_{S,\mathrm{DEP}}$ can be realized. Finally, the amplified CDS signal is stored in the memory cell via the memory SF M_3_.

The single transistor amplification allows for a low noise linear operation, since it does not add any noise to the sampled charge in principle [36]. It is measured showing less than 3% non-linearity at 500 mV input swing [37]. Meanwhile, the NMOS-only structure avoids the use of PMOS fitting with the pixel concept and the existing CDS scheme at a small area cost without excessive controls. Additionally, the amplification alone does not require DC biasing current, which reduces power consumption and hence less generated heat in the pixel. Due to the presence of the parasitic capacitance *C*_p_, only a gain of about 3 can be obtained in practice. It is still sufficient for the noise reduction as discussed earlier in Section 2.

### 3.3. CDS Amplification with Additional CDS SF Stage (Type B)

**Figure 6:**
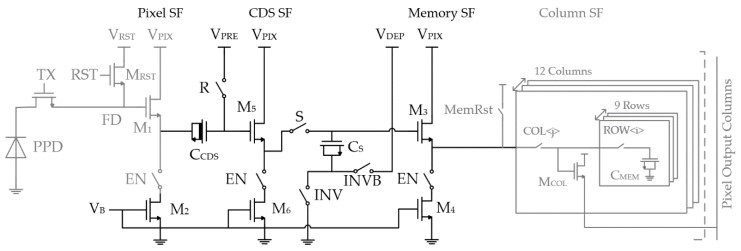
Pixel schematic (Type B).

In Type A, $C_{\mathrm{CDS}}$ must be sized multiple times greater than $C_{S}$, which brings several drawbacks. First, for a fixed $A_{\mathrm{CDS}}$, $C_{S}$ is not allowed to be sized larger independently for better noise performance, as $C_{\mathrm{CDS}}$ must follow. Secondly, it is not practical to improve the gain beyond 0.8, as a much greater $C_{\mathrm{CDS}}$ (more than 4 times) is needed. Thirdly, a high $C_{\mathrm{CDS}}$ degrades the transient performance, since a longer tracking time is required for the pixel SF. Based on Type A, Type B is proposed as highlighted in Figure 6. An extra CDS SF stage M_5_ is inserted between $C_{\mathrm{CDS}}$ and $C_{S}$, which can avoid the capacitive division between them.

The additional buffer between $C_{\mathrm{CDS}}$ and $C_{S}$ allows independent optimization for each of them, which brings several benefits. First, the new CDS stage gain is mainly defined by the CDS SF gain, which is typically slightly lower than 1 due to the body effect. Without sacrificing the gain, $C_{\mathrm{CDS}}$ is free to be sized smaller in favor of faster settling. Secondly, a higher $C_{S}$ can be used, leading to lower sampling noise. Additionally, the passive amplification gain is improved because the impact of parasitic capacitance is less pronounced. Although an additional CDS SF stage is added in Type B, to ensure a fair comparison, both pixels are designed with the same total power budget by setting different SF biasing currents. Consequently, the current sources are tailored to adapt the changes.

### 3.4. Noise Analysis of the Proposed Pixels

To quantitatively evaluate the total noise performance, the noise models for Type A and Type B are presented in this section. Given the targeted operation frequency of 20 MHz, only the thermal noise sources are taken into account in the noise analysis, as thermal noise is assumed to be the dominant noise source at this bandwidth [21].

#### 3.4.1. Noise Model on the Type A Pixel

**Figure 7:**
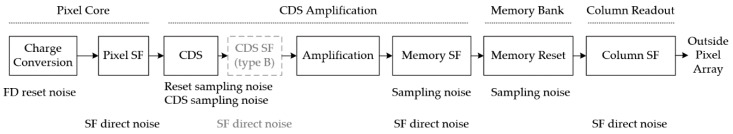
Noise diagram overview based on pixel operations. In the Type B pixel, there is an additional source follower (SF) direct noise due to the CDS SF.

Based on the pixel operation flow, the noise block diagram is presented in Figure 7. The noise contributors can be separated into four stages: the pixel core, the CDS amplifier, the memory bank, and the column readout. Those noises can be sorted into two categories: the kTC noise due to the sampling switches as the sampling noise and the noise from the SF stages as the SF direct noise. Since they are uncorrelated noise sources, the total noise pixel noise can be considered as the sum of these two types of noises from different stages. Assuming these noises are stationary, each of the noise components will be modeled by integrating the noise power spectral density (PSD) [38].

Taking Type A as example, the first noise contributor is the FD node reset noise. The reset noise can be modeled as $kT/C_{\mathrm{FD}}$, where $C_{\mathrm{FD}}$ represents the total capacitance associated with the FD node. When this noise is fully settled, it can be canceled completely with the in-pixel CDS technique. However, because the pixel SF M_1_ is sized very small for high CG, during the short tracking phase, it is difficult to achieve perfect settling on the large RST switching transient (typically a few hundred millivolts). As a result, a small amount of the reset noise $V_{n,\mathrm{FD}}$ may remain in the output. Simplifying the pixel SF stage as a single pole system, the input referred noise residue can be characterized as

(3) $$V_{in,\mathrm{kTC}\;\mathrm{RES}}^{2}=\frac{kT}{C_{\mathrm{FD}}}e^{-\frac{2T_{\mathrm{TR}}\left(g_{m1}+g_{\mathrm{Load}}\right)}{C_{\mathrm{CDS}}}}$$

where $T_{\mathrm{TR}}$ is the tracking phase time, $g_{m1}$ is the transconductance of M_1_, and $g_{\mathrm{Load}}$ is the output load of the pixel SF stage. It suggests that a longer tracking time, a high $g_{m1}$, or a smaller $C_{\mathrm{CDS}}$ can improve the CDS efficiency.

**Figure 8:**
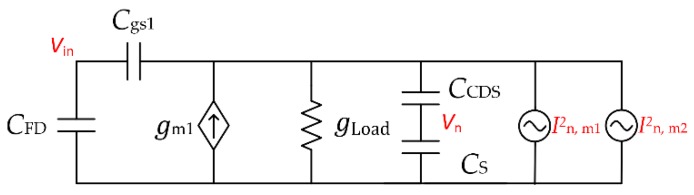
Small signal model for pixel SF direct noise.

Secondly, when S is OFF, the pixel SF noise is directly sampled on $C_{S}$. Its corresponding small signal model is shown in Figure 8. The capacitance $C_{\mathrm{gs}1}$ is the M_1_ gate-to-source capacitance. The $I_{n,m1}$ and $I_{n,m2}$ are thermal current noise sources from the transistor M_1_ and M_2_, respectively, which can be modeled as $I_{n,m1}^{2}=4kT\gamma g_{m1}$ and $I_{n,m2}^{2}=4kT\gamma g_{m2}$, where γ is the transistor’s thermal noise coefficient and $g_{m2}$ is the transconductance of M_2_. Thus, the FD node input referred noise spectrum from these noise sources can be calculated as

(4) $$V_{\mathrm{in},\mathrm{PIX}\;\mathrm{SF}}^{2}\left(s\right)=\frac{4kT\gamma \left(g_{m1}+g_{m2}\right){\left(C_{\mathrm{gs}1}+C_{\mathrm{FD}}\right)}^{2}}{A_{\mathrm{PIX},\mathrm{SF}}^{2}{\left(C_{\mathrm{gs}1}g_{\mathrm{Load}}+C_{\mathrm{FD}}g_{m1}+C_{\mathrm{FD}}g_{\mathrm{Load}}+C_{\mathrm{eq}}g_{\mathrm{gs}1}s+C_{\mathrm{eq}}C_{\mathrm{FD}}s+C_{\mathrm{gs}1}C_{\mathrm{FD}}s\right)}^{2}}$$

where $C_{\mathrm{eq}}$ is the equivalent the capacitance of $C_{\mathrm{CDS}}$ and $C_{S}$ in series. By integrating the noise spectrum over the full bandwidth, as $C_{\mathrm{FD}}$ and $C_{\mathrm{gs}1}$ are much smaller, the temporal pixel SF input referred noise can be calculated and further simplified as

(5) $$V_{\mathrm{in},\mathrm{PIX}\;\mathrm{SF}}^{2}=\;\frac{kT\gamma \left(g_{m1}+g_{m2}\right)\left(C_{\mathrm{gs}1}+C_{\mathrm{FD}}\right)}{A_{\mathrm{PIX},\mathrm{SF}}^{2}A_{\mathrm{CDS}}C_{S}\left(g_{m1}+g_{\mathrm{Load}}\right)C_{\mathrm{FD}}}.$$

Here $C_{\mathrm{eq}}$ is replaced with $A_{\mathrm{CDS}}C_{S}$ for better insight. Intuitively, increasing $A_{\mathrm{CDS}}$ or enlarging the size of $C_{S}$ can reduce the temporal noise, since the thermal noise is limited by the circuit bandwidth.

The CDS operation will introduce additional kTC noise, when the reset signal and CDS signal are sampled. The sum of these two can be modeled as

(6) $$V_{\mathrm{in},\mathrm{CDS}\;\mathrm{RST}}^{2}+V_{\mathrm{in},\mathrm{CDS}\;\mathrm{SAMP}}^{2}=\frac{kT}{A_{\mathrm{PIX},\mathrm{SF}}^{2}}\left(\frac{C_{\mathrm{CDS}}+C_{S}}{C_{\mathrm{CDS}}^{2}}+\frac{{\left(C_{\mathrm{CDS}}+C_{S}\right)}^{2}}{C_{\mathrm{CDS}}^{2}C_{S}}\right).$$

Compared to kTC noises generated at the FD node and memories, the CDS noise can be made much smaller by making $C_{\mathrm{CDS}}$ greater. Given a total area budget of $C_{\mathrm{CDS}}+C_{S}$, the optimal $C_{\mathrm{CDS}}$ budget ratio between those can be found mathematically by minimizing Equation (6), which shares the same expression as the $A_{\mathrm{CDS}}$:

(7) $$\mathrm{Optimal}\;A_{\mathrm{CDS}}=\frac{C_{\mathrm{CDS}}}{C_{\mathrm{CDS}}+C_{S}}=\frac{7-\sqrt{17}}{4}\approx 0.72.$$

The noises in the memory bank include the memory sampling, the memory SF M_3_ and the memory column reset operation, which can be similarly calculated accordingly as

(8) $$V_{\mathrm{in},\mathrm{MEM}}^{2}+V_{\mathrm{in},\mathrm{MEM}\;\mathrm{SF}}^{2}+V_{\mathrm{in},\mathrm{MEM}\;\mathrm{RST}}^{2}=\frac{1}{A_{\mathrm{PIX},\mathrm{SF}}^{2}A_{\mathrm{AMP}}^{2}A_{\mathrm{MEM},\mathrm{SF}}^{2}A_{\mathrm{CDS}}^{2}}\frac{kT}{C_{\mathrm{MEM}}}+\frac{kT\gamma \left(g_{m3}+g_{m4}\right)}{\left(g_{m3}+g_{\mathrm{Load}\;\mathrm{MEM}}\right)\left(C_{\mathrm{MEM}}+C_{p}\right)}+\frac{C_{P}kT}{C_{\mathrm{MEM}}^{2}}$$

where $A_{\mathrm{MEM},\mathrm{SF}}$ is the memory SF gain, $g_{m3}$ and $g_{m4}$ are the transconductances of the corresponding transistors, $g_{\mathrm{LoadMEM}}$ is the intrinsic output load of the memory SF, and $C_{P}$ is the parasitic capacitance at the gate of M_3_. Note that, for simplicity, the gate-to-source capacitance and the gate-to-drain capacitance of M_3_ are neglected as they are much smaller compared to $C_{\mathrm{MEM}}$. As expected, the signal gain prior to the memory bank has a significant impact on the input referred noise stemming from the memory bank. Without the additional amplifier gain $A_{\mathrm{AMP}}$, the noise from the memory storage can easily become dominant. The balance between the noise from memory bank and the noise from added CDS amplification is the key trade-off in our pixel architecture.

The last noise contributor is the column SF. Benefiting from the low operation speed and pico-farad sampling capacitors, its direct noise is negligible compared to the rest. Therefore, the total input referred noise for Type A pixel can be approximated based on the above equations.

#### 3.4.2. Noise Model on the Type B Pixel

A similar noise model is developed for Type B. Compared to Type A, the major difference is the CDS operation noise due to the extra SF stage. With the additional CDS SF, including M_5_ and M_6_, the new sampling noise from the Type B can be modeled as

(9) $$V_{\mathrm{in},\mathrm{CDS}\;\mathrm{RST}}^{2}+V_{\mathrm{in},\mathrm{CDS}\;\mathrm{SAMP}}^{2}=\frac{kT}{A_{\mathrm{PIX},\mathrm{SF}}^{2}}\left(\frac{1}{C_{\mathrm{CDS}}}+\frac{1}{A_{\mathrm{CDS},\mathrm{SF}}^{2}C_{S}}\right)$$

where $A_{\mathrm{CDS},\mathrm{SF}}$ is the gain of CDS SF. Assuming a typical SF gain $A_{\mathrm{CDS},\mathrm{SF}}$ is 0.9, the optimal theoretical $C_{\mathrm{CDS}}$ budget ratio for the Type B can be found as 0.47 by minimizing Equation (9). It confirms that, while maintaining a similar CDS stage gain, $C_{\mathrm{CDS}}$ can be sized smaller, leading to a better settling. Benefiting from it, the current of the pixel SF stage can be adjusted so that Type B pixel can still have the same power consumption even with the extra CDS SF. Of course, it comes at the cost that the CDS SF brings additional direct noise that can be modeled as

(10) $$V_{\mathrm{in},\mathrm{CDS}\;\mathrm{SF}}^{2}=\frac{1}{A_{\mathrm{PIX},\mathrm{SF}}^{2}A_{\mathrm{CDS},\mathrm{SF}}^{2}}\left(\frac{kT\gamma \left(g_{m5}+g_{m6}\right)}{\left(g_{m5}+g_{\mathrm{LoadCDS}}\right)C_{S}}\right)$$

where $g_{m5}$ and $g_{m6}$ are the transconductances of the corresponding transistors and $g_{\mathrm{LoadCDS}}$ is the intrinsic output load of the CDS SF. Similarly, the gate-to-source capacitance and the gate-to-drain capacitance of M_5_ are neglected. As $C_{S}$ can be sized independently from $C_{\mathrm{CDS}}$, its added noise can be easier to control.

#### 3.4.3. Noise Analysis and Comparison

**Figure 9:**
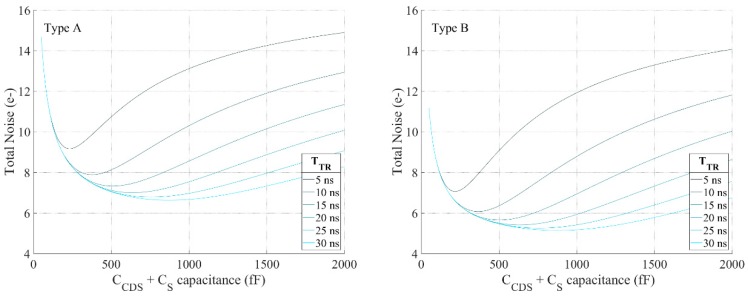
The input referred noise against sum of $C_{\mathrm{CDS}}$ and $C_{S}$ w.r.t to different tracking time (15 ns settling time is targeted for 20 Mfps operation). **Left**: Type A. **Right**: Type B. The $C_{\mathrm{CDS}}$ and $C_{S}$ are chosen according to the optimal ratio based on Equations (7) and (9).

The noise model can provide valuable insights on the design constraints for further optimizations. As discussed, the capacitances $C_{\mathrm{CDS}}$ and $C_{S}$ are very important for the noise and speed. When the total available CDS area is too small, the kTC noise from the CDS operation will dominate the overall noise; when it is too large, the transient kTC residue noise becomes more influential as well. Therefore, there is an optimal value for $C_{\mathrm{CDS}}$ and $C_{S}$. Using the noise models and simulation parameters the optimal $C_{\mathrm{CDS}}$ and $C_{S}$ sizes can be found for both types as shown in Figure 9. To eliminate the kTC residue noise, 15 ns of reset tracking time is chosen. Thus, in both types, the CDS operation contributes more noise compared to the reset noise residue. Limited by the available area and considering that more blocks can benefit from a higher gain for Type A, 200 fF and 50 fF capacitors were finally chosen for $C_{\mathrm{CDS}}$ and $C_{S}$, respectively. On the right figure, Type B shows a better noise performance at the same area budget. Due to the overhead of the CDS SF, parasitics of the CDS SF and the layout constraints, 100 fF and 75 fF were implemented for $C_{\mathrm{CDS}}$ and $C_{S}$, respectively.

**Figure 10:**
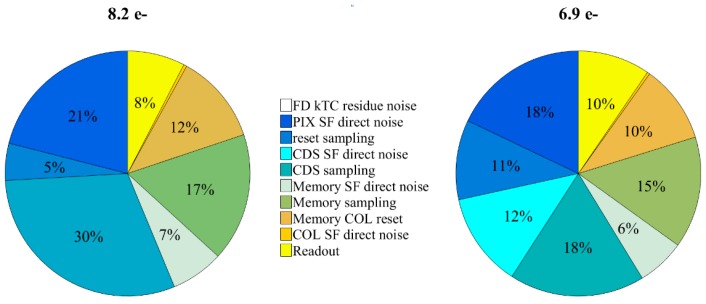
The total input referred noise power breakdown for Type A (**left**) and Type B (**right**). The input referred photon noise is calculated based on the conversion gain of 100 µV/e^−^.

Including the noise from the rest of the readout blocks of our test system, the total calculated input referred noise power break-down for the two pixel types is presented in Figure 10. Both types are designed with the same total area and power consumption. With the help of in-pixel amplification, the analytical total input referred noise of Type A is found to be 8.2 e^−^, while it is 6.9 e^−^ in Type B. The kTC residue noise is negligible for both cases which is consistent with Figure 9. As a reference, the 10 fF memory cell alone can generate kTC noise of 630 µV. In the case of no in-pixel amplification, even with a unity gain buffer, the input referred noise of memory kTC is already 6.3 e^−^. Compared to it, both Type A and Type B show a significant improvement on the memory sampling noise, as the noise from the memory bank only contributes 17% and 15% of the total noise, respectively. It also confirms that, since the memory and the column SF are no longer the major noise contributors, higher amplification gain is indeed marginal in improving the noise.

Compared to Type A, Type B shows an overall better noise performance and the noise is more widely distributed over different noise components. The top contribution of CDS sampling is reduced from 30 to 18% thanks to a greater $C_{S}$ and a higher amplification gain. Considering the initial overhead of implementing the additional CDS SF, Type B is preferred to be implemented for larger pixels.

## 4. Measurement Results and Discussion

**Figure 11:**
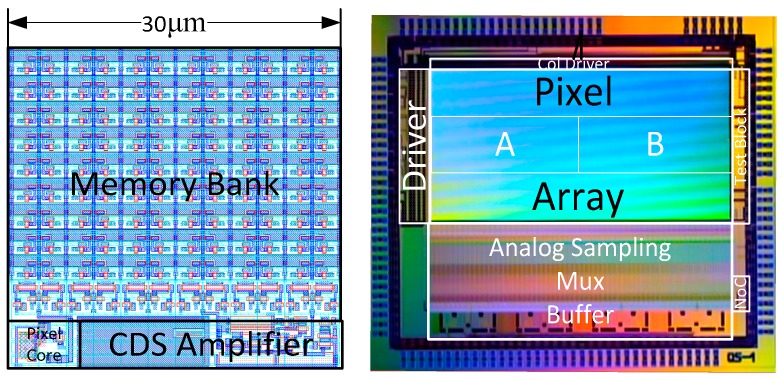
The Type A pixel layout and the chip microphotograph.

The proposed pixel concept is designed and fabricated as two 32 × 84 pixel arrays (one for each type) in imec’s 130 nm CMOS CIS technology. As an example, the complete layout of the Type A pixel is shown in the left side of Figure 11. The pixel core occupies an area of 5 × 5 µm^2^, which is kept away from the remaining pixel circuitry for less parasitics and interference. The CDS amplification stage is located next to the pixel core occupying an area of 25 × 5 µm^2^. The rest of the pixel area is covered by the memory bank and its column SFs.

As shown in the chip microphotograph on the right side of Figure 11, the pixel drivers are located on the left side of the pixel array as well as on the top for driving the memory bank columns. Since the prototype has a small pixel count, the column readout block is not optimized for high repetitive rates in the burst mode readout. When the pixel signals are ready on the column lines, they are sampled in the analog sampling stage. Via the analog mux, eight analog output buffers are used to transfer the signals to off-chip ADCs. The converted digital data is then collected and transferred to the PC by using a custom FPGA board.

The pixel performance is characterized with a homogeneous illumination LED light source. The photon transfer curve (PTC) measurements [39] were evaluated over different frame rates to confirm the viability of both Type A and Type B pixels. To mimic a mechanic shutter and reduce the parasitic light, the LED light source is switched off during the long readout time to prevent an undesired light collection. The PTC is then measured by only varying the LED light intensity with the same integration time. This ensures the true high-speed operation with the shortcoming that our LED light source is not powerful enough to saturate the pixels at very high frame rates.

**Figure 12:**
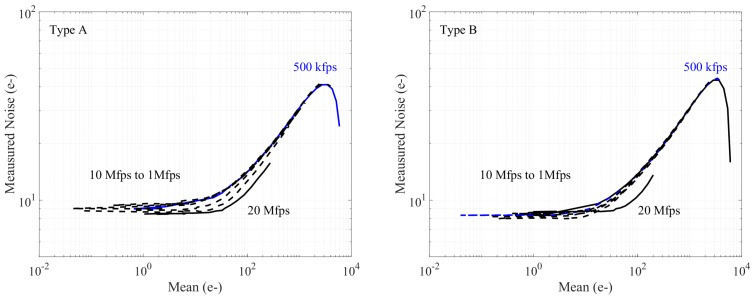
The PTCs for Type A and Type B measured at speeds from 500 kfps to 20 Mfps. Different curves present different operation frequencies. Due to the limited LED power, only the pixels at the 500 kfps case can reach the saturation region.

The PTC measurement results for both pixel types are shown in Figure 12, where the measured output noise has been referred to the FD node and converted to electrons based on its pixel conversion gain. Most of the PTC curves are close to each other over different frequencies. Both 20 Mfps cases are, however, slightly deviating, which may be a result of the non-linearity at such a speed. The total conversion gain was extracted based on the 500 kfps case, in which the pixels were finally saturated. The extracted total input referred noises are 9 e^−^ and 8.4 e^−^ for Type A and Type B, respectively, including the noise of additional readout blocks (analog samplers, buffers, and off-chip ADCs). The measured input referred noises are higher than these expected, which can be explained by the full chain gain difference between the models and the measurements. Compared to model values, the measured gains are 13% and 16% lower for Type A and Type B, respectively. Given that there are multiple gain stages in the signal chain, a small inaccuracy in each stage can be easily accumulated. As expected, the passive amplification in Type B shows a higher gain, which is extracted as 3.4 compared to 2.9 in Type A. This confirms that the parasitic capacitance plays a key role in the single transistor passive amplification.

**Figure 13:**
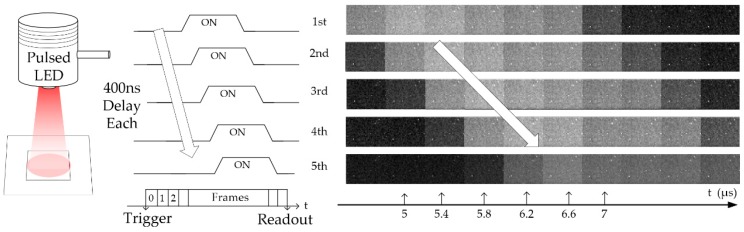
Pulsed light measurement at 5 Mfps. Each light pulse is triggered with a fixed delay of 400 ns following the previous one. The same delay can be confirmed from the sensor measurement.

A light measurement was also done by using a pulsed LED light source. By delaying the trigger time of the LED light pulse in each burst capture, a series of images at 5 Mfps operation is obtained, which is shown in Figure 13, demonstrating the functionality of the test imager at high speed.

For burst mode imagers, the signals will stay in memory up to a few milliseconds. By sweeping the readout time, based on the memory size, the leakage current on the 10 fF memory cells is measured as 4.6 nA/cm^2^ referred to the pixel size, measured at ambient temperature of 24 °C without cooling. In another word, it takes more than 160 ms for the leakage/dark current to change the signal for 5 mV, which is still sufficiently long for the slow readout in this prototype.

## 5. Conclusions

This paper presents a novel pixel architecture for CMOS burst mode ultra-high-speed imagers, which is enabling the low noise operation at a high frame depth. Equipped with a 108-cell memory bank inside the 30 µm pitch pixel, a prototype imager with two flavors of CDS amplification stages is demonstrated. Moreover, analytical noise models are provided and allow for a better design trade-off. By using the NMOS-only in-pixel CDS and amplification, a low noise of 8.4 e^−^ is measured with a small 10 fF memory cell, showing a good trade-off between the noise and the frame depth. Table 1 summarizes the test chip performance. For large imager formats (e.g., VGA), due to the high pixel current and the large pixel pitch, IR drop and control signal integrity will become very challenging. Since the 3D technology becomes available, the in-situ memory bank can potentially be integrated on the top of the pixel array, which will be the next milestone for future burst mode imagers.

## 6. Patents

The resulted work has been granted as US Patent Number 9756270.

## Figures and Tables

**Table 1:** Test imager specification summary.

Technology	130 nm CMOS CIS	Full Well Capacity ^1^	6 ke^−^
Pixel size	30 µm	Conversion gain	105 µV/e^−^
Memory depth	108 (10 fF/cell)	Burst rate	20 Mfps
Array size	32 × 84 (for Type A/B)	Power	10 µA/pixel @ 3.3 V(for Type A/B)
Dynamic range (@500 kfps)	56 dB (Type A) 57 dB (Type B)	Read noise	9 e^−^ (Type A) 8.4 e^−^ (Type B)

^1^ FWC is defined by the maximum saturation level.
